# The Combination of DAT-SPECT, Structural and Diffusion MRI Predicts Clinical Progression in Parkinson’s Disease

**DOI:** 10.3389/fnagi.2019.00057

**Published:** 2019-03-15

**Authors:** Sara Lorio, Fabio Sambataro, Alessandro Bertolino, Bogdan Draganski, Juergen Dukart

**Affiliations:** ^1^Developmental Neurosciences, UCL Great Ormond Street Institute of Child Health, University College London, London, United Kingdom; ^2^Roche Pharma and Early Development, Neuroscience, Ophthalmology and Rare Diseases, F. Hoffmann-La Roche Ltd., Basel, Switzerland; ^3^Laboratory for Research in Neuroimaging, Department of Clinical Neurosciences, Lausanne University Hospital, University of Lausanne, Lausanne, Switzerland; ^4^Department of Experimental and Clinical Medical Sciences, University of Udine, Udine, Italy; ^5^Department of Basic Medical Science, Neuroscience and Sense Organs, University of Bari, Bari, Italy; ^6^Max Planck Institute for Human Cognitive and Brain Sciences, Leipzig, Germany; ^7^Institute of Neuroscience and Medicine, Brain and Behaviour (INM-7), Research Centre Jülich, Jülich, Germany; ^8^Institute of Systems Neuroscience, Medical Faculty, Heinrich Heine University Düsseldorf, Düsseldorf, Germany

**Keywords:** Parkinson’s disease, voxel-based morphometry, voxel-based quantification, covariance analysis, symptoms severity

## Abstract

There is an increasing interest in identifying non-invasive biomarkers of disease severity and prognosis in idiopathic Parkinson’s disease (PD). Dopamine-transporter SPECT (DAT-SPECT), diffusion tensor imaging (DTI), and structural magnetic resonance imaging (sMRI) provide unique information about the brain’s neurotransmitter and microstructural properties. In this study, we evaluate the relative and combined capability of these imaging modalities to predict symptom severity and clinical progression in *de novo* PD patients. To this end, we used MRI, SPECT, and clinical data of *de novo* drug-naïve PD patients (*n* = 205, mean age 61 ± 10) and age-, sex-matched healthy controls (*n* = 105, mean age 58 ± 12) acquired at baseline. Moreover, we employed clinical data acquired at 1 year follow-up for PD patients with or without L-Dopa treatment in order to predict the progression symptoms severity. Voxel-based group comparisons and covariance analyses were applied to characterize baseline disease-related alterations for DAT-SPECT, DTI, and sMRI. Cortical and subcortical alterations in *de novo* PD patients were found in all evaluated imaging modalities, in line with previously reported midbrain-striato-cortical network alterations. The combination of these imaging alterations was reliably linked to clinical severity and disease progression at 1 year follow-up in this patient population, providing evidence for the potential use of these modalities as imaging biomarkers for disease severity and prognosis that can be integrated into clinical trials.

## Introduction

Parkinson’s disease (PD) is primarily characterized by progressive accumulation of aggregated α-synuclein in the brainstem, leading to a degeneration of dopaminergic neurons in substantia nigra (Lang and Lozano, 1998; Xu et al., 2002; Braak et al., 2003; Kraemmer et al., 2014). This presumably toxic accumulation induces a progressive loss of dopaminergic input to the striatum and further degeneration of striato-cortical pathways, resulting in the occurrence of different motor and non-motor symptoms (Houk and Wise, 1995; Lang and Lozano, 1998; Kraemmer et al., 2014). Current PD drugs focus on the symptoms treatment, however, the main goal of pharmaceutical research is to develop drugs able to slow or even stop the clinical progression.

To improve the monitoring of disease progression and the evaluation of drug effectiveness, it is essential to identify biomarkers able to detect the neurodegenerative alterations at all circuitry levels. Such biomarkers should demonstrate a strong and reproducible correlation with pathological changes and symptoms severity in multicenter studies (McGhee et al., 2013).

Several imaging modalities have been suggested for that purpose in the literature. Dopamine transporter single photon emission tomography (DAT-SPECT) provides a semi-quantitative assessment of striatal dopaminergic deafferentation. It is a well-established diagnostic biomarker of PD, owing to the strong correlation between the amount of dopamine transporters in the striatum and the number of dopaminergic neurons in substantia nigra (Kraemmer et al., 2014). However, DAT-SPECT does not provide information on non-dopaminergic disease aspects, and its link to disease progression in *de novo* PD patients remains unclear (Kägi et al., 2010). Structural MRI (sMRI) and diffusion tensor imaging (DTI) are powerful tools to assess whole brain atrophy patterns and microstructural tissue integrity (Le Bihan et al., 2001; Ashburner et al., 2003; Burton et al., 2004). Several sMRI studies reported volumetric changes in PD using voxel-based morphometry (VBM) analysis (Burton et al., 2004; Nagano-Saito et al., 2005; Wattendorf et al., 2009). However, only few studies reported the association between volumetric changes or cortical thinning and PD symptoms severity, measured by neuropsychological scores for testing attention and memory, and olfactory alterations (Junqué et al., 2005; Camicioli et al., 2011; Melzer et al., 2012; Segura et al., 2014; Campabadal et al., 2017). Also microstructural brain changes assessed through DTI using mean diffusivity (MD) and fractional anisotropy (FA) maps were found in PD throughout different brain regions (Gattellaro et al., 2009; Peran et al., 2010; Wang et al., 2011; Zhang et al., 2011, 2015; Du et al., 2012; Zhan et al., 2012; Kim et al., 2013; Schwarz et al., 2013; Tan et al., 2015; Lim et al., 2016; Loane et al., 2016; Nagae et al., 2016). However, most of these studies measured structural changes in small, heterogeneous PD patient cohorts (e.g., highly varying disease duration), resulting in variable magnitude and directionality of the respective findings (McGhee et al., 2013; Meijer et al., 2013; Weingarten et al., 2015). Moreover single DTI measures failed to accurately assess disease severity and prognosis, hampering their application in standard clinical practice or as biomarkers in clinical trials (McGhee et al., 2013; Pyatigorskaya et al., 2014; Atkinson-Clement et al., 2017). In contrast, in a small proof-of-concept study in PD patients, multimodal MRI measures based on DTI and R2^∗^ maps have been shown to provide complementary information allowing for a better differentiation of patients and controls (Peran et al., 2010). However, the relative and combined value of MRI measures and DAT-SPECT as biomarkers of disease severity and prognosis remains unknown.

Despite imaging changes in individual brain regions can be used as diagnostic biomarker, theories on neuro-degenerative disease increasingly focus on the role of brain network alterations as potential predictors of early disease severity and prognosis (Helmich et al., 2010; Woo et al., 2017). In fact previous studies showed that PD targets regions that, in healthy individuals have a well-defined correlation patterns (Helmich et al., 2010; Woo et al., 2017). However, the interaction between changes in the striatal dopamine and the morphological and microstructural alterations in the striato-cortical circuits remains unclear. This interaction can be evaluated using structural covariance analysis, which examines whether an imaging measure in one region correlates with the variation of the same, or other modalities measures in other brain regions (Mechelli et al., 2005; Schmitt et al., 2009; Alexander-Bloch et al., 2013a). Complementary to functional MRI (fMRI) and DTI-based connectomics, population covariance in brain anatomy represents another source of information about inter-regional anatomical associations (Alexander-Bloch et al., 2013b). A crucial difference between fMRI, diffusion MRI networks, and structural covariance is that the latter estimates inter-regional correlations based on group of images, while the first two networks can be constructed from connectivity measures computed for an individual image (Alexander-Bloch et al., 2013a,b). Another challenge of the structural covariance networks might be the biological interpretation of the results (Alexander-Bloch et al., 2013a). However, recent studies showed that the structural covariance pattern are influenced by synaptic connectivity between brain regions, genetic and developmental relationships, and different degenerative processes (Gong et al., 2012; Chou et al., 2015; Chang et al., 2017; de Schipper et al., 2017; Oosterwijk et al., 2018; Yee et al., 2018). Therefore, we applied this method to investigate structural network alterations using MRI-based measurements and striatal dopamine transporter uptake derived from DAT-SPECT.

Here, we evaluated DAT-SPECT, DTI and sMRI as biomarkers of disease severity and progression in *de novo* PD patients. Disease severity was assessed longitudinally through the modified version of the unified Parkinson’s disease rating scale (MDS-UPDRS) evaluated at baseline (same time point at which the imaging data was acquired) and at 1 year follow-up. We used well-established VBM, voxel-based quantification (VBQ) (Draganski et al., 2011) and structural covariance analyses to identify disease-related alterations in a large cohort of *de novo* PD patients. We then tested whether combinations of imaging alterations identified in drug-naïve *de novo* PD were associated with current clinical severity measured by baseline MDS-UPDRS scores. Moreover, we employed those imaging measure to predict the future disease progression quantified by the MDS-UPDRS variations between the two time points. As the dopaminergic treatments taken by some patients after the baseline evaluation have substantial effects on the clinical symptoms measured with MDS-UPDRS, we accounted for those effects by applying the prediction models separately to the group of patients receiving medication and to the one without treatment employing off medication clinical assessment (Mueller et al., 2018; van Ruitenbeek et al., 2018).

## Materials and Methods

### Subjects

Data of *de novo* PD patients (*N* = 205) and healthy controls (HC) (*N* = 105) included in this study were obtained from the Parkinson’s Progression Markers Initiative (PPMI) database ^1^. PPMI is a large multicenter study and each site independently received ethics approval of the protocol. This study was carried out in accordance with Good Clinical Practice (GCP) regulations and International Conference on Harmonization (ICH) guidelines. Written informed consent was obtained from all participants in accordance with the Declaration of Helsinki.

All subjects underwent sMRI, DTI, and DAT-SPECT at baseline, and had MDS-UPDRS 1, 2, 3, and total, acquired at baseline (BL) and after 1 year (TP1). *De novo* PD patients included in the PPMI database had a diagnosis confirmed by DAT-SPECT scans and Hoehn and Yahr stage I or II. The exclusion criteria were diagnosis of dementia, psychiatric disorders or other neurological disease detectable with MRI at baseline. At BL all patients were drug-naïve, while at TP1 some patients started drug treatment with L-Dopa, dopamine agonist, and other unspecified medications. Only categorical (yes/no) information was recorded on the respective treatment categories (L-Dopa, dopamine agonists or other PD medication) without information about the dosage. In the current study, we included patients remaining without drug treatment at TP1 (called as PD no med) and patients taking only L-Dopa treatment at TP1 (called as PD on med). The PD on med group was composed of patients having the MDS-UPDRS3 evaluation performed off medication (patients were asked not to take L-Dopa for 24 h before the evaluation). From the 205 patients, we identified 56 subjects for the PD no med group, and 44 subjects for the PD on med group.

### Image Acquisition and Processing

Whole-brain MRI was performed using standardized protocols on different 3T scanners. All acquisition protocols included a standard T1-weighted MPRAGE sequence (TI/TR = 900/2,300 ms, TE = 2.98 ms, 1 mm isotropic resolution), and a 2D single-shot echo-planar DTI sequence for diffusion weighted images (TR/TE = 900/88 ms, 2 mm isotropic resolution, diffusion weighting along 64 gradient directions, b-value = 1000 s/mm^2^).

DAT-SPECT images were obtained using different camera systems. Details about SPECT image reconstruction are available on the PPMI website (see footnote 1) (reconstructed image matrix 91 × 109 × 91, 2 mm isotropic resolution).

VBM analysis was performed on the T1-weighted images using automated tissue classification and enhanced subcortical tissue probability maps embedded into the unified segmentation framework of SPM12 (Ashburner and Friston, 2005; Lorio et al., 2016). Image registration to the MNI space was performed for each subject applying subject-specific diffeomorphic estimates obtained using DARTEL (Ashburner, 2007) with default settings on the gray and white matter tissue maps (respectively, GM and WM). The warped GM maps were scaled by the Jacobian determinants of the deformation fields to account for local compression and expansion due to linear and non-linear transformation (Ashburner and Friston, 2000), resulting in GM volume maps. The GM volume maps were then smoothed using an isotropic Gaussian kernel of 6 mm full width at half maximum (FWHM).

The DTI maps were calculated from the diffusion weighted data using a group of libraries called TEEM^2^. The pre-processing steps for the estimation of FA and MD maps included correction for distortions due to eddy currents and head motion (Rohde et al., 2004; Tao et al., 2009), and affine registration of the corrected diffusion weighted data to the T1-weighted images using FLIRT from FSL5.0 ^3^ (Greve and Fischl, 2009).

For VBQ analysis, FA and MD maps were warped into MNI space using a non-linear registration approach based on the subject-specific diffeomorphic estimates (Ashburner, 2007), derived for the GM and WM maps without scaling by the Jacobian determinants. In order to enhance the specificity of the warped FA and MD values for brain tissue classes, we used the combination of weighting procedure with the GM and WM probability maps derived from the T1-weighted data, and Gaussian smoothing with a 6 mm FWHM isotropic smoothing kernel as described by Draganski et al. (2011). Separate FA and MD maps were generated for GM and WM sub-spaces.

DAT-SPECT data pre-processing was performed within SPM12 and included normalization to an average size DAT-SPECT template with subsequent normalization into the MNI space performed using the “normalization” function. Then the images were non-linearly warped from the MNI to the native space of the T1-weighted data using the spatial transformation parameters estimated for the GM and WM probability maps. This allowed the correction for partial volume effects using the modified Müller-Gartner method (Müller-Gärtner et al., 1992; Rousset et al., 1998) based on the convolution of the DAT-SPECT data with the tissue classification maps estimated from T1-weighted images. The GM SPECT images were then normalized to MNI space using parameters derived from T1-weighted data and scaled to the global mean GM signal for each subject. Finally, the SPECT images were smoothed with an isotropic Gaussian kernel of 6 mm FWHM.

### Statistical Analysis and Voxel-Based Group Comparison

We used odds ratios and *t*-test, as implemented in Matlab 2012b, to assess gender and age differences between PD patients and HC, and between PD on med and PD no med groups (Table 1). We compared the values of MDS-UPDRS scales (1, 2, 3, and total) measured at BL, TP1 and the differences between the two time points, using two-sample *t*-tests between the respective groups (see Table 1). Only MDS-UPDRS 3 values measured off medication were compared at TP1. Fisher’s exact test was used to compare the disease dominant side between PD on med and PD no med subgroups (see Table 1).

**Table 1:** Demographic and clinical data of subjects included in the study.

Demographic		HC	PD	PD no med	PD on med	PD versus HC *p*-value	PD on med versus PD no med *p*-value
	
		Mean ± SD					
Number of subjects (m/f)		105 (74/31)	205 (143/62)	56 (34/22)	44 (28/16)	0.999	0.837
Age		58 ± 12	61 ± 10	59 ± 10.2	62.6 ± 10.1	0.207	0.084
MDS-UPDRS1	Baseline	2 ± 1	6 ± 4	5 ± 3	6 ± 4	**<10^−6^** ^∗^	**0.022**^∗^
	Follow-up	–	–	6 ± 4	7 ± 4	–	**0.030 ^∗^**
	Change	–	–	1 ± 2	2 ± 3	–	**0.041^∗^**
MDS-UPDRS2	Baseline	0.1 ± 0.03	6 ± 4	6 ± 3	7 ± 4	**<10^−6^** ^∗^	**0.012**^∗^
	Follow-up	–	–	7 ± 4	8 ± 4	–	**0.021^∗^**
	Change	–	–	2 ± 2	3 ± 2	–	**0.031^∗^**
MDS-UPDRS3	Baseline	0.6 ± 0.3	21 ± 9	19 ± 10	22 ± 9	**<10^−6^^∗^**	0.121
	Follow-up	–	–	21 ± 11	24 ± 10	–	0.067
	Change	–	–	4 ± 9	5 ± 10	–	0.067
MDS-UPDRS total	Baseline	3 ± 1.8	33 ± 13	29 ± 10	34 ± 15	**<10^−6^** ^∗^	**0.031**^∗^
	Follow-up	–	–	32 ± 15	37 ± 16	–	0.069
	Change	–	–	7 ± 10	8 ± 9	–	0.100
Dominant side (left/right/equilateral)		–	78/117/10	20/35/1	20/22/2	–	0.309

*HC, healthy controls; PD, Parkinson’s disease patients, PD no med, patients without medications 1 year after baseline; PD on med, patients on L-Dopa treatment 1 year after the baseline. Bold characters and “^∗^” indicate a significant group difference (p < 0.05).*

To test for local differences in GM volume maps, FA and MD between PD patients and HC, we used SPM12 to perform a voxel-wise t-statistic separately for every map and tissue class (GM and WM), using a flexible factorial design, controlling for sex, age, total intracranial volume (TIV) and image acquisition site. These tests were carried out using explicit masks defining GM and WM voxels. The masks were generated as follows: smoothed (FWHM of 6 mm isotropic), Jacobian-modulated tissue probability maps in MNI space were averaged across all subjects for each tissue class (GM and WM). Then binary masks were generated by applying a threshold of 20%, voxels for which neither the GM nor the WM probability exceeded that value were excluded from the analysis. This approach was used to ensure that each voxel was analyzed in only one subspace and that non-brain tissue was excluded.

Similarly, differences in DAT-SPECT were evaluated using voxel-based comparisons, restricting analyses to GM voxels and controlling for age and sex. We applied to all voxel-wise statistical analyses a family-wise error (FWE) corrected cluster threshold of *p* < 0.05 combined with a voxel-wise *p* < 0.001. We further estimated the effect size (Cohen’s *d*) of the observed differences.

To test for differential interaction between striatal dopamine transporter uptake derived from DAT-SPECT and MRI-based measurements, we performed voxel-wise covariance analyses (Mechelli et al., 2005) between DAT-SPECT values in the putamen (region showing strongest differences between PD patients and HC) and GM volume, FA and MD maps. The putamen was delineated according to the basal ganglia human area template (Prodoehl et al., 2008). We computed separate covariance analyses for each MRI-based measure (GM volume, FA, MD) and each tissue type (gray and white matter for FA and MD), controlling for age, sex, TIV, disease dominant side and image acquisition site. The covariance analyses were carried out using GM and WM masks obtained as previously described.

An FWE-corrected cluster threshold of *p* < 0.05 combined with a voxel-wise *p* < 0.001 was applied for all analyses. To quantify the differential inter-modality correlations identified in the covariance analyses, we computed Spearman correlation coefficients between the putamen DAT-SPECT signal and the average value of the age and sex adjusted MRI-based measures showing significant between-group differences in the covariance analysis.

### Prediction Models: Symptoms Severity and Disease Progression

To evaluate the combined value of imaging measures as biomarkers of symptoms severity we used multiple linear regression models to predict each MDS-UPDRS subscale. The MDS-UPDRS 1, 2, 3, and total were the dependent variables, while the models regressors were the mean imaging measures extracted from regions showing significant differences between PD patients and HC (group differences: FA in pons nuclei, prefrontal MD, DAT signal in putamen; covariance differences: prefrontal GM volume, brainstem MD). For regions identified in covariance analyses we included in the model an interaction term between the MRI measure and DAT-SPECT. All analyses were controlled for age, sex, and symptoms dominant side. The goodness of fit for each model was assessed using the determination coefficient (*R*^2^), while the model overfit was evaluated by means of the leave-one-out adjusted *R*^2^ coefficient. Additionally, we estimated the Pearson correlation coefficient between each of the above-mentioned imaging measures and the MDS-UPDRS 1, 2, 3, and total separately.

To test if the BL imaging measures were prognostic biomarkers of disease progression, we used multiple linear regression models for predicting the changes in MDS-UPDRS scores (ΔMDS-UPDRS) between TP1 and BL. The ΔMDS-UPDRS 1, 2, 3, and total were the dependent variables, while the model regressors were the FA values in pons nuclei, prefrontal MD, DAT signal in putamen, and the interaction between putamen DAT signal and prefrontal GM volume, brainstem MD, respectively. The longitudinal prediction models were computed using data of both PD no med and on med groups. Patients receiving dopamine agonists at TP1 were not included in this analysis due to the longer wash out time of those treatments and their potential effect on the off medication assessment of symptoms severity.

To account for the effect of L-Dopa treatment on the prediction models, we performed separate regression analyses for the PD on med and no med groups. Demographic and clinical details of the two subgroups are provided in Table 1. Moreover we computed the Pearson correlation coefficient between each of the imaging measures and the ΔMDS-UPDRS 1, 2, 3, and total separately for the PD on med and no med groups.

The multiple regression models for each MDS-UPDRS and ΔMDS-UPDRS subscales were considered as significant at *p* < 0.05. In case of a significant multiple regression model, single regressors were considered as significant at *p* < 0.01. The Pearson correlation coefficients were corrected for multiple comparison using the false discovery rate (FDR) at *q* < 0.05.

## Results

### Demographic and Clinical Variables

Patients and HC were matched for gender and age, as shown in Table 1. The groups PD on med and PD no med did not differ with respect to sex, age, and disease dominant side (see Table 1). All clinical scores were significantly higher for patients as compared to HC (Table 1). The group PD on med showed significantly higher MDS-UPDRS 1, 2, and total scores at BL and TP1, as well as higher ΔMDS-UPDRS1 and ΔMDS-UPDRS2 in comparison to the PD no med group (Table 1). No significant differences were found for the MDS-UPDRS3 at BL, TP1, and ΔMDS-UPDRS3 between PD on med and no med groups.

### Voxel-Based Group Comparison

**FIGURE 1:**
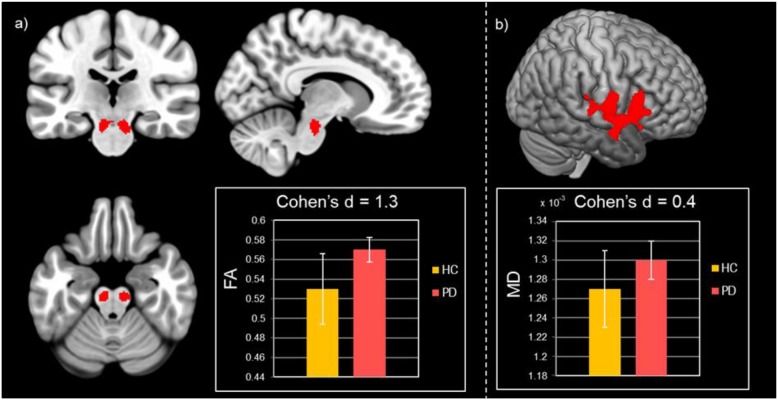
Results of group comparisons for diffusion MRI measures. **(a)** Increased FA values in Parkinson’s disease patients compared to healthy controls at pFWE < 0.05. **(b)** Higher MD values for Parkinson’s disease patients with respect to healthy controls at pFWE < 0.05.

In PD patients significantly higher FA values were found in the brainstem WM corresponding to the pontine tegmentum (Table 2 and Figure 1a). Increased MD values were found in patients with respect to HC in the operculum (Table 2 and Figure 1b). No significant differences between patients and HC were observed for GM volume.

**Table 2:** Main differences for group comparisons and covariance analyses done at baseline between healthy controls (HC) and Parkinson’s disease patients (PD).

Analysis	Region	Coordinates (mm)			*T*-value	Cluster size
		*x*	*y*	*z*		
FA maps: PD > HC	Left pontine tegmentum	−8	−21	−21	4.7	163
	Right pontine tegmentum	9	−22	−22	4.5	167
MD maps: PD > HC	Right operculum	58	9	−2	5	1571
Covariance DAT-MD: PD > HC	Pons nuclei	4	−26	−28	4	884
Covariance DAT-GM volume: PD > HC	Left prefrontal cortex	−44	20	34	4.6	681
	Left premotor cortex	−8	6	39	4.5	607
	Left insula	−54	−14	16	4	625
	Right insula	58	−12	14	4	686

**FIGURE 2:**
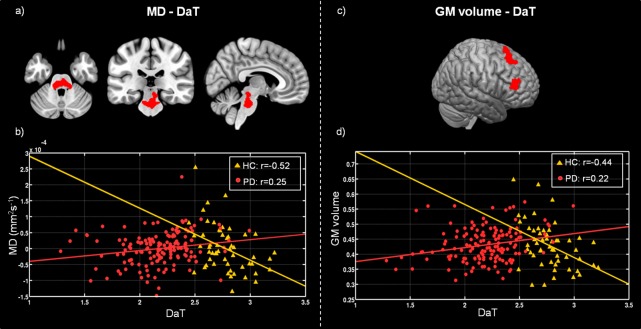
Differences between healthy controls (HC) and Parkinson’s disease patients (PD) in voxel-wise correlation of MD maps with DAT-SPECT measured in putamen **(a)**, and GM volume with mean DAT-SPECT in putamen **(c)**. **(b)** Scatter plot showing the correlation between the DAT-SPECT measured in the putamen and the mean MD values computed within the region highlighted in red on panel **(a)**, for HC (yellow triangles) and PD (red circles). **(d)** Scatter plot showing the correlation between the DAT-SPECT measured in the putamen and the mean GM volume estimated within the region highlighted in red on panel **(c)**, for HC (yellow triangles) and PD (red circles). Plot legend reports Spearman’s correlation coefficients.

In the structural covariance analyses, we found significant differences in the correlation between DAT-SPECT in the putamen and MD values in the pons nuclei across patients and HC. The Spearman correlation coefficients showed a significant positive correlation in PD patients (ρ = 0.25; *p* < 0.001), and a significant negative one in HC (ρ = −0.52; *p* < 0.001) (Figure 2a,b).

Furthermore, we found significant differences between patients and HC in the covariance analysis evaluating the association between DAT-SPECT signal in the putamen and GM volume in the left prefrontal, premotor cortex and in the insula. We observed a significant positive correlation (ρ = 0.22; *p* < 0.001) for patients, and a significant negative correlation for controls (ρ = −0.44; *p* < 0.001) (Figure 2c,d). No significant differences were found for structural covariance between DAT-SPECT and FA values across groups.

### Prediction Models: Symptoms Severity and Disease Progression

The linear combinations of DAT-SPECT values in the putamen, and MRI differences found in the group comparison, significantly predicted the BL MDS-UPDRS2 and MDS-UPDRS total but not the other subscales (see Tables 3, 4). The MD values in the operculum, the DAT-SPECT signal in the putamen and age were the most significant predictors for the MDS-UPDRS2, as summarized in Tables 3, 4. The significant predictors for the MDS-UPDRS total were the MD values in the operculum, the putamen DAT-SPECT values, and the interaction between putamen DAT-SPECT – GM volume in premotor, prefrontal cortex and insula (see Tables 3, 4).

**Table 3:** Summary of the linear models explaining the MDS-UPDRS components at baseline (BL) and the MDS-UPDRS variation (ΔMDS-UPDRS) 1 year after the baseline.

Time point	Dependent variable	Model predictors: *p*-value										*R*^2^	Adj *R*^2^	*F*-, *p*-value
		GM vol in PFC and PMC	MD in OP	MD in PN	FA in PT	DAT in Put	Interaction DAT in Put – MD PN	Interaction DAT in Put – GM vol in PFC and PMC and IN	Age	Gender	Dominant side			
BL	MDS-UPDRS1	0.37	0.11	0.75	0.81	0.35	0.05	0.01	0.37	0.9	0.83	0.12	0.09	0.82, 0.57
	**MDS-UPDRS2**	**0.30**	**0.01^∗^**	**0.08**	**0.17**	**−2 × 10^−4^^∗^**	**0.39**	**0.10**	**0.01^∗^**	**0.87**	**0.12**	**0.38**	**0.29**	**4, 2 × 10^−4∗^**
	MDS-UPDRS3	0.88	0.09	0.40	0.85	0.50	0.06	0.11	0.02	0.13	0.22	0.21	0.09	2, 0.08
	**MDS-UPDRS tot**	**0.9**	**0.002^∗^**	**0.23**	**0.50**	**0.006^∗^**	**0.03**	**0.002^∗^**	**0.01**	**0.31**	**0.70**	**0.31**	**0.21**	**4.85, 0.003^∗^**
Δ	MDS-UPDRS1 PD on med+no med	0.78	0.29	0.25	0.44	0.37	0.74	0.58	0.13	0.35	0.34	0.15	0.03	1.02, 0.43
	**MDS-UPDRS1 PD on med**	**0.87**	**0.70**	**0.003^∗^**	**0.47**	**0.01^∗^**	**0.002^∗^**	**0.32**	**0.008^∗^**	**0.96**	**0.87**	**0.47**	**0.27**	**5.7, 0.004^∗^**
	MDS-UPDRS1 PD no med	0.28	0.11	0.44	0.38	0.5	0.9	0.21	0.44	0.09	0.06	0.19	0.01	1, 0.41
	MDS-UPDRS2 PD on med+no med	0.02	0.30	0.36	0.03	0.03	0.03	0.80	0.01	0.12	0.18	0.22	0.09	1.71, 0.09
	**MDS-UPDRS2 PD on med**	**0.006^∗^**	**0.009^∗^**	**0.17**	**0.01^∗^**	**0.004^∗^**	**0.25**	**0.004^∗^**	**0.66**	**0.05**	**0.27**	**0.37**	**0.15**	**4.68, 0.01^∗^**
	**MDS-UPDRS2 PD no med**	**0.003^∗^**	**0.02**	**0.09**	**0.01^∗^**	**0.01^∗^**	**0.23**	**0.01^∗^**	**0.55**	**0.6**	**0.11**	**0.37**	**0.21**	**4.75, 0.01^∗^**
	MDS-UPDRS3 PD on med+no med	0.02	0.96	0.94	0.01	0.03	0.41	0.20	0.41	0.22	0.25	0.15	0.05	1.1, 0.38
	MDS-UPDRS3 PD on med	0.76	0.74	0.47	0.34	0.95	0.74	0.48	0.77	0.99	0.44	0.27	0.13	0.59, 0.83
	MDS-UPDRS3 PD no med	0.61	0.19	0.9	0.5	0.6	0.02	0.09	0.87	0.28	0.7	0.20	0.03	1.14, 0.35
	MDS-UPDRS tot PD on med+no med	0.02	0.98	0.92	0.01	0.01	0.03	0.71	0.08	0.98	0.38	0.17	0.03	1.3, 0.31
	MDS-UPDRS tot PD on med	0.82	0.58	0.69	0.58	0.5	0.06	0.61	0.79	0.58	0.38	0.21	0.05	0.89, 0.54
	MDS-UPDRS tot PD no med	0.14	0.24	0.03	0.34	0.04	0.73	0.37	0.86	0.16	0.22	0.27	0.11	1.69, 0.11

*Models for the ΔMDS-UPDRS were estimated separately for the patients without medication 1 year after baseline (no med), for the patients on L-Dopa 1 year after baseline (on med) and for the patients of both groups together (on med + no med). The model predictors were the mean imaging values extracted from the following regions: IN, insula; OP, operculum; PFC, prefrontal cortex; PMC, premotor cortex; PN, pons nuclei; Put, putamen; PT, pontine tegmentum. We report the p-value for every regressor, and the determination coefficient (R^2^), adjusted R^2^, f-value, and p-value for every model. Bold characters and “^∗^” indicate a significant (p < 0.05) model – (p < 0.01) regressors.*

**Table 4:** Summary of the slopes (β) of the linear regressions explaining the MDS-UPDRS components at baseline (BL) and the MDS-UPDRS variation (ΔMDS-UPDRS) 1 year after the baseline.

Time point	Dependent variable	Model predictors: β slope									
		GM vol in PFC and PMC	MD in OP	MD in PN	FA in PT	DAT in Put	Interaction DAT in Put – MD PN	Interaction DAT in Put – GM vol in PFC and PMC and IN	Age	Gender	Dominant side
BL	MDS-UPDRS1	2.8	−3.7	104.7	−1.8	8.6 × 10^4^	−3.1 × 10^3^	−9.4	10^4^	0.1	−0.1
	**MDS-UPDRS2**	−**0.54**	−**19.8**	**50.2**	−**6.5**	**3.4 × 10^4^**	−**1.5 × 10^4^**	**9.8**	**1.1 × 10^4^**	−**0.1**	−**0.7**
	MDS-UPDRS3	0.8	−6.7	149	−2.9	1.9 × 10^5^	−1.9 × 10^4^	3.7	2.6 × 10^4^	0.23	3.3
	**MDS-UPDRS tot**	**3**	−**30.3**	**303**	−**11.2**	**3.1 × 10^5^**	**3.8 × 10^4^**	**4**	**4.8 × 10^4^**	**0.36**	**3.1**
Δ	MDS-UPDRS1 PD on med+no med	−2	−9.1	19.2	−0.4	−3.2 × 10^4^	−8.5 × 10^3^	3.5	5.1 × 10^3^	0.1	−1.1
	**MDS-UPDRS1 PD on med**	−**3.4**	**23.2**	**81.4**	−**1.9**	−**2.3 × 10^5^**	−**2.6 × 10^4^**	−**8.3**	−**3.1 × 10^3^**	**0.1**	−**1.4**
	MDS-UPDRS1 PD no med	1.2	−14.5	−63.4	−1.3	5.6 × 10^3^	−7.2 × 10^3^	11.5	1.1 × 10^4^	0.04	−1.7
	MDS-UPDRS2 PD on med+no med	2.3	−1	−29.7	−0.6	−7.5 × 10^4^	6.4 × 10^3^	7.6	1.7 × 10^4^	−0.01	0.5
	**MDS-UPDRS2 PD on med**	−**9.4**	−**20.1**	**235**	**9.3**	**3.3** ×**10^4^**	**3.2** ×**10^4^**	−**24.1**	**1.5** ×**10^4^**	**0.07**	**2.9**
	**MDS-UPDRS2 PD no med**	**5.5**	−**26.8**	−**116.8**	−**2.8**	−**5.9** ×**10^4^**	−**865.3**	**9.5**	**2.3** ×**10^4^**	−**0.03**	−**0.5**
	MDS-UPDRS3 PD on med+no med	9.5	29.6	14.1	−1.3	−2.2 × 10^5^	1.1 × 10^4^	7.2	2.1 × 10^4^	−0.1	−1
	MDS-UPDRS3 PD on med	−5	−42.8	447.3	13.6	3.3 × 10^5^	1.1 × 10^5^	−139.7	1.2 × 10^4^	−0.03	2.7
	MDS-UPDRS3 PD no med	9.8	−31	−240.1	−2.9	−3.1 × 10^5^	−4.2 × 10^3^	15.2	2.5 × 10^4^	0.02	−3
	MDS-UPDRS tot PD on med+no med	9.8	19.5	3.7	−2.3	−3.3 × 10^5^	9.1 × 10^3^	18.3	4.3 × 10^4^	−0.01	−1.6
	MDS-UPDRS tot PD on med	−17.7	−40	763.8	21.1	1.3 × 10^5^	1.1 × 10^5^	−172.1	2.4 × 10^4^	0.1	4.1
	MDS-UPDRS tot PD no med	16.5	−72.3	−420.3	−7.1	−3.6 × 10^5^	−1.2 × 10^4^	36.1	5.9 × 10^4^	0.03	−5.3

*Models for the ΔMDS-UPDRS were estimated separately for the patients without medication 1 year after baseline (no med), for the patients on *L*-Dopa 1 year after baseline (on med) and for the patients of both groups together (on med + no med). The model predictors were the mean imaging values extracted from the following regions: IN, insula; OP, operculum; PFC, prefrontal cortex; PMC, premotor cortex; PN, pons nuclei; Put, putamen; PT, pontine tegmentum. Bold characters indicate a significant (*p* < 0.05) model – (*p* < 0.01) regressors.*

We did not find any significant correlation between the single imaging measures and the BS MDS-UPDRS scores.

For the longitudinal prediction models we found that the combination of BL DAT-SPECT and MRI measures was not able to significantly predict the ΔMDS-UPDRS scores for the group of patients with and without medication (see Tables 3, 4).

However the linear combination of BL imaging measures significantly predicted ΔMDS-UPDRS2 for the PD no med group, and ΔMDS-UPDRS1 and 2 for the PD on med (Tables 3, 4). The significant regressors for the prediction of ΔMDS-UPDRS2 in the PD no med group were GM volume in the premotor and prefrontal cortex, putamen DAT-SPECT, FA in the pontine tegmentum and the interaction between DAT signal in the putamen – GM volume in premotor, prefrontal cortex and insula (Tables 3, 4). For the PD on med, the significant predictors of ΔMDS-UPDRS1 were the MD in the pons nuclei, the DAT-SPECT in the putamen, interaction DAT – MD in the pons nuclei and age. The premotor and prefrontal GM volume, putamen DAT binding, MD in the pons nuclei and the interaction DAT in putamen – MD in pons nuclei significantly predicted ΔMDS-UPDRS2 for the PD on med group.

We did not find any significant correlation between the single imaging measures and the ΔMDS-UPDRS scores for both the PD no med and on med groups.

## Discussion

Here, we evaluated the relative and combined value of MRI and DAT-SPECT measures as biomarkers of disease severity and clinical progression in *de novo* PD patients. Consistent with prior literature, we identified DAT-SPECT and MRI abnormalities in patients compared to the HC (Brooks, 1998; Lee et al., 2000; Isaias et al., 2007; Gattellaro et al., 2009; Wang et al., 2011). Most importantly, we found that in combination, these imaging abnormalities could reliably predict both current clinical symptoms and their progression over time, suggesting that they could be used as prognostic biomarkers.

### Clinical Variables

Prior to perform the image comparison and the prediction analysis of the clinical scores, we statistically compared the MDS-UPDRS across groups. As expected, we found significantly higher clinical scores for the PD patients with respect to the HC. Moreover, we observed significantly higher increase of the MDS-UPDRS 1, 2, and total scores at BL and TP1, as well as ΔMDS-UPDRS1 and ΔMDS-UPDRS2 for the PD group on med with respect to the PD no med group. Those results are in agreement with the fact that patients on medication exhibit more advanced disease stages. The lack of significant difference in MDS-UPDRS3 between the PD on med and PD no med groups might be explained by the fact that the motor assessment is a highly variable measure, involving both patient- and rater-dependent variability (Mentzel et al., 2016; Rahmim et al., 2017). The use of a longitudinal clinical assessment over 1 year might have increased this variability, confounding potential differences between the groups.

### Regional Findings

Earlier studies have reported altered diffusion MRI measures across different brain areas in PD and associated animal models (Peran et al., 2010; Wang et al., 2011; Zhang et al., 2011, 2015; Du et al., 2012; Schwarz et al., 2013; Tan et al., 2015; Lim et al., 2016; Loane et al., 2016; Nagae et al., 2016). Consistent with those findings, we showed distinct patterns of MD alterations in the prefrontal cortex, suggesting underlying microstructure degradation that might be due to the loss of dopaminergic input into the striatum (Hornykiewicz, 1998; Mattay et al., 2002; Gattellaro et al., 2009; Zhan et al., 2012; Kim et al., 2013).

Furthermore, we found increased FA values in PD patients in midbrain regions corresponding to the location of pontine tegmentum. This region is affected by the alpha synuclein pathology at early disease stages (Braak et al., 2003; Mori et al., 2008; Venda et al., 2010), and it is associated with the patho-physiology of sleep behavior disorder frequently observed at prodromal disease stage (Iranzo et al., 2006; Burke and O’Malley, 2013). There is substantial controversy in the literature about the magnitude, directionality and neurobiological interpretation of FA changes in this region. While Wang et al. (2011) also found increased FA values in the substantia nigra, other studies reported lowered FA in PD patients in this region (Peran et al., 2010; Rolheiser et al., 2011; Du et al., 2012; Prakash et al., 2012; Zhang et al., 2016). The reason for this divergence remains unclear but might be related to the inclusion of more advanced PD patients in those studies.

### Structural Network Findings

In agreement with previous research showing 40–60% loss of striatal dopaminergic innervations from substantia nigra in *de novo* PD patients, we found a significantly reduced DAT-SPECT signal in the striatum (Brooks, 1998; Lee et al., 2000; Isaias et al., 2007). Our findings on significant interactions between putamen DAT uptake and MD values in the pons nuclei are in line with the known disruption of brainstem projections to the striatum. More specifically, we found that a lower striatal DAT uptake was linked to lower MD values in PD patients, which was the opposite of what we observed in HC. This finding supports the idea that structural covariance might be more sensitive to axonal and neuronal damage in the ponto-mesencephalic tegmentum, which did not show significant MD changes in direct group comparisons. The pons nuclei represent the anatomical origin of projections modulating the dopaminergic action of the substantia nigra and they are under dopaminergic inhibitory control from the ventral tegmental area, which is known to degenerate in PD (Guiard et al., 2008). According to the physiological functions attributed to the noradrenergic system, impaired functioning of pons nuclei in PD results primarily in affective disorders (Remy et al., 2005), cognitive disturbances (Javoy-Agid and Agid, 1980), sleep disorders (Boeve et al., 2007), sensory impairment (Braak et al., 2003) and autonomic dysfunction (Orskov et al., 1987).

Furthermore, in PD patients we found a decrease in putamen DAT being linked to lower GM volume values in the premotor-prefrontal cortex, while in HC a reduction in putamen DAT was associated to a bigger GM volume. While, we did not detect any significant atrophy in those cortical regions using group comparisons, these findings suggested that progressive loss of striatal DAT uptake translates into GM volume loss in premotor-prefrontal regions. This result is in line with studies showing that significant GM volume loss is predominantly observed in more advanced PD patients exhibiting cognitive deficits (Beyer et al., 2007; Melzer et al., 2012). The clinical stage arises when the disease spreads from brainstem to basal ganglia nuclei and then to cortical regions in an ascending course (Lang and Lozano, 1998; Braak et al., 2003).

### Prediction of Symptom Severity and Disease Progression

After identifying these alterations in patients compared to HC, we then evaluated their link to current disease severity and their prognostic value for future symptoms progression measured by the MDS-UPDRS subscales. We showed that the combination of the imaging measures was significantly related to the current clinical severity measured by MDS-UPDRS2 and MDS-UPDRS total. The imaging measures that better predicted the BL MDS-UPDRS2 were the MD values in the operculum and the DAT-SPECT signal in the putamen, while the MDS-UPDRS total was better predicted by the MD values in the operculum, the putamen DAT-SPECT values, and the interaction between putamen DAT-SPECT – GM volume in premotor, prefrontal cortex and insula. The absence of significant correlation between each of those imaging measures and the MDS-UPDRS2 and MDS-UPDRS total is in agreement with results previously reported literature (McGhee et al., 2013). Moreover this result highlights the fact that the combination of those imaging measures, rather than each single one, could be used as biomarker for predicting symptoms measured by MDS-UPDRS2.

The MDS-UPDRS2 is a subscale evaluating the subject’s impairment in daily life activities (Goetz et al., 2008), and it is often used to evaluate symptoms severity perceived by the patient. Consequently MDS-UPDRS2 can be used to assess improvements related to pharmacological treatments or surgical therapies such as deep brain stimulation (Rodriguez-Oroz et al., 2005).

One key objective of the study was to evaluate the association between the imaging biomarkers and disease progression. As dopaminergic treatments have substantial effects on the clinical symptoms measured with MDS-UPDRS, those treatments represent a potential confound that needs to be controlled for. Due to this reason we first performed the predictive analysis of symptoms progression using the entire PD cohort and then we employed separate models for the PD no med and on med groups. For the patients on medications we employed the MDS-UPDRS scores assessed off medication. The off medication clinical assessments performed for the PPMI study were acquired asking the patients not to take PD medication the day before. While, the half-life of L-Dopa is short enough to achieve a sufficient wash out in 24 h, the half-life of dopamine agonists or other medications could be longer and therefore be not sufficient to estimate a proper off medication symptom severity (Brooks, 2008; Onofrj et al., 2009; Reichmann, 2009). For this reason, we restricted our analyses of the patients treated only with L-Dopa.

We found that GM volume extracted from the premotor cortex, putamen DAT, the interaction between both, and brainstem FA values were significant predictors for ΔMDS-UPDRS2 in the untreated patients. The ΔMDS-UPDRS2 in the group on medication was significantly predicted by the same measures with the addition of MD assessed in the pons nuclei. Though the separation of PD patients in no med and on med groups was performed to exclude confounds of treatment onto the MDS-UPDRS scales, the separate analyses could be also considered as independent replication samples of the prognostic link of MRI and DAT measures onto clinical symptoms measures. This consistency of the significant association between the identified structural measures and ΔMDS-UPDRS2 across both groups strengthens the confidence in these findings.

Finally, we found that the MD in the pons nuclei, putamen DAT-SPECT, interaction DAT – MD in the pons nuclei were significant predictors of ΔMDS-UPDRS1, which is associated to non-motor and cognitive deficits. However, the significant prediction of the ΔMDS-UPDRS1 was only observed in the on med group. Beside the possibility of being a false positive finding, this difference could also reflect the more severe phenotype in the on med group or the more complex effects of treatment onto clinical severity captured by MDS-UPDRS1. In fact the L-Dopa treatment of the on med group is likely to interfere with the clinical measures of symptoms severity as no off medication assessment was performed for the MDS-UPDRS1. Therefore it is important to replicate this association in an independent cohort in order to evaluate the prognostic ability of those imaging measures for the ΔMDS-UPDRS1.

The linear combination of DAT-SPECT and MRI measures was not able to significantly predict the symptoms progression at TP1 using the data of both PD no med and on med groups. This results might be indicative of the fact that using heterogeneous groups of patients could confound the reliability of predictive analysis (Miller and O’Callaghan, 2015; Tuite, 2016).

The lack of significant correlation between each of the imaging measures used in this study and the ΔMDS-UPDRS scores confirms previous literature results (McGhee et al., 2013). The crucial finding of this analysis was that the combination of DAT-SPECT imaging, structural and diffusion MRI achieved reliable prediction of some symptom severity scores and disease progression in 1 year in homogeneous cohort of patients, as reported by a previous study (Rahmim et al., 2017), while the single imaging measures failed to provide significant correlation with those scores. Many studies in literature assessed the correlations between disease severity-progression and brain changes in PD patients using uni-modal approaches (Burton et al., 2004; Nagano-Saito et al., 2005; Benninger et al., 2009; Wattendorf et al., 2009; Schwarz et al., 2013). The results cover different regions with a wide variety of repeatability and low correlations with clinical scores.

The findings obtained in the current study can be indicative of the presence of microstructural tissue changes induced by the disease in the early stages. In light of the correlation with the clinical progression, the combination of the imaging measures affected by those tissue changes could be employed as disease progression biomarker.

Cross-sectional and longitudinal studies in PD patients will be crucial to confirm our results and establish the value of the identified imaging alterations in more advanced and prodromal PD populations.

### Limitations and Outlook

Here, we investigate the association between different imaging and clinical measures in a large cohort of *de novo* untreated PD patients. The acquisition of all those imaging modalities might be time-consuming and expensive, limiting their use in clinical protocols. However, recent advances in MRI hardware, such as improved scanner gradient performance, and software, such as parallel imaging and multi-band imaging sequences (Griswold et al., 2002; Setsompop et al., 2012; Uecker et al., 2014), can substantially reduce the time required for acquiring MRI data.

Despite the good correlation between brain changes and clinical scores, the study provides a reliable prediction only for some symptom subtypes. This might be due to the fact that the disease progression differentially affects the various cortical-subcortical circuits (Kish et al., 1988).

We find several potential structural and molecular imaging biomarkers altered in *de novo* PD patients. We further show the potential of these alterations as biomarkers of current symptom severity and their prognostic value with respect to evolution of specific PD symptom domains.

## Data Availability

Publicly available datasets were analyzed in this study. This data can be found here: https://www.ppmi-info.org/.

## Author Contributions

JD, FS, AB, and BD conceptualized the research project. JD organized the research project. SL executed the research project and statistical analysis, and wrote the first draft of manuscript. SL and JD designed the statistical analysis. JD critically reviewed the statistical analysis. SL, JD, FS, AB, and BD critically reviewed the manuscript.

## Conflict of Interest Statement

This study was sponsored by F. Hoffmann-La Roche, Basel, Switzerland. The authors received no specific funding for this work. F. Hoffmann-La Roche provided financial contribution in the form of salary for SL, FS, AB, and JD but did not have any additional role in the study design, data collection and analysis, decision to publish, or preparation of the manuscript. The remaining author declares that the research was conducted in the absence of any commercial or financial relationships that could be construed as a potential conflict of interest.

## Footnotes

1 www.ppmi-info.org

2 http://teem.sourceforge.net/index.html

3 www.fmrib.ox.ac.uk/fsl
